# Expression profiling of *TaARGOS* homoeologous drought responsive genes in bread wheat

**DOI:** 10.1038/s41598-022-07637-y

**Published:** 2022-03-04

**Authors:** Kashif Ahmed, Ghulam Shabbir, Mukhtar Ahmed, Sabahat Noor, Atta Mohi Ud Din, Maqsood Qamar, Nazia Rehman

**Affiliations:** 1 Pir Mehr Ali Shah Arid Agriculture University, Rawalpindi, Pakistan; 2 National Agricultural Research Centre (NARC), Islamabad, Pakistan; 3 College of Agriculture, Nanjing Agricultural University, Najing, China

**Keywords:** Biological techniques, Biotechnology, Chemical biology, Genetics, Plant sciences, Plant biotechnology, Plant breeding, Plant cell biology, Plant development, Plant molecular biology, Plant physiology, Plant stress responses

## Abstract

Drought tolerant germplasm is needed to increase crop production, since water scarcity is a critical bottleneck in crop productivity worldwide. Auxin Regulated Gene involved in Organ Size (*ARGOS*) is a large protein family of transcription factors that plays a vital role in organ size, plant growth, development, and abiotic stress responses in plants. Although, the *ARGOS* gene family has been discovered and functionalized in a variety of crop plants, but a comprehensive and systematic investigation of *ARGOS* genes in locally used commercial wheat cultivars is still yet to be reported. The relative expression of three highly conserved *TaARGOS* homoeologous genes (*TaARGOS-A, TaARGOS-B, TaARGOS-D)* was studied in three drought-tolerant (Pakistan-2013, NARC-2009 and NR-499) and three sensitive (Borlaug-2016, NR-514 and NR-516) wheat genotypes under osmotic stress, induced by PEG-6000 at 0 (exogenous control), 2, 4, 6, and 12 h. The normalization of target genes was done using *β-actin* as endogenous control, whereas *DREB3*, as a marker gene was also transcribed, reinforcing the prevalence of dehydration in all stress treatments. Real-time quantitative PCR revealed that osmotic stress induced expression of the three *TaARGOS* transcripts in different wheat seedlings at distinct timepoints. Overall, all genes exhibited significantly higher expression in the drought-tolerant genotypes as compared to the sensitive ones. For instance, the expression profile of *TaARGOS-A* and *TaARGOS-D* showed more than threefold increase at 2 h and six to sevenfold increase after 4 h of osmotic stress. However, after 6 h of osmotic stress these genes started to downregulate, and the lowest gene expression was noticed after 12 h of osmotic stress. Among all the homoeologous genes, *TaARGOS-D*, in particular, had a more significant influence on controlling plant growth and drought tolerance as it showed the highest expression. Altogether, *TaARGOSs* are involved in seedling establishment and overall plant growth. In addition, the tolerant group of genotypes had a much greater relative fold expression than the sensitive genotypes. Ultimately, Pakistan-2013 showed the highest relative expression of the studied genes than other genotypes which shows its proficiency to mitigate osmotic stress. Therefore, it could be cultivated in arid and semi-arid regions under moisture-deficient regimes. These findings advocated the molecular mechanism and regulatory roles of *TaARGOS* genes in plant growth and osmotic stress tolerance in contrasting groups of wheat genotypes, accompanied by the genetic nature of identified genotypes in terms of their potential for drought tolerance.

## Introduction

Increasing crop productivity has been the main objective of the breeding program due to an upsurge in the demand and use of agricultural commodities for feed, food, and fuel, following substantial growth in the global population and emerging economies^1–4^. Increases in crop yield can be attributable to higher total biomass productivity (larger plants produce more yield), a better harvest index, or even both^3,5^. Plants have a well-known and complex regulatory system to protect their growth, production, and development. Many abiotic stresses, such as drought, salinity, and high temperatures, have severe consequences on certain plant parts. Plants respond to environmental challenges by inhibiting or stimulating the expression of many genes with specialized functions to flourish and grow^6,7^. Gene expression profiling under particular circumstance helps to determine the particular genes that are being expressed in a cell. There are several experiments that can measure the entire genome with the use of this approach^8^. This can be done by comparing mRNA levels in two or more experimental circumstances, and then determining which conditions induced the expression of specific genes^9^. In this perspective, transcription factors play a crucial role in the signal transformation networks by regulating gene expression. They turn on or off-target gene expression directly, disrupting the interplay of many gene signaling networks^2,10–13^. As a result, crop breeding should focus on genes that regulate organ size and contribute to better production^14^.

The ORGAN SIZE RELATED (*OSR*) family of genes is recognized to enhance plant organ growth and standardize gene expression of organ size^13,15–17^. Additionally, earlier research has revealed that the *OSR* gene family is implicated in the reaction to abiotic stress^16,18–20^. The founding member of the *OSR* homologs, AUXIN REGULATED GENE INVOLVED IN ORGAN SIZE (*ARGOS*), has been identified in Arabidopsis as an auxin-induced gene that is transcribed in growing organs. Overexpression of *AtARGOS*, which encodes a putative integral membrane protein, is ample to enhance organ size by stimulating proliferation of cells; downregulation, on the other hand, causes decreased organ growth. Furthermore, In Arabidopsis, four *OSR* homologs and other members of this family (*OSR1* and *OSR2*) were found to drive the development of plant organs when overexpressed^13,17^. Five members of *OSR* have been recognized in rice, comprising *OsARGOS*^17^. In Arabidopsis, the overexpression of *OsARGOS* was found to support organ growth^1,21^. Maize has eight copies of the *ARGOS* gene. The maize plant organ growth and yield are progressed by the overexpression of *ZmARGOS1*^20^, while abiotic stress has also been linked to *ARGOSs* in maize crop^18,19^. Overexpression of *ZmARGOS1* and *ZmARGOS8* in Arabidopsis and maize enhances drought tolerance through an ethylene-dependent regulatory mechanism. In addition, in both dry and hydrated conditions, the over expressive *ZmARGOS8* maize plants produce more grains than wild-type^19^. Furthermore, *DREB3s* (dehydration responsive element binding) are also pivotal transcription factors in plant species. These transcription factors regulate expression in an ABA-independent pathway of many stress-inducible genes and playing an important role in improving plant drought tolerance^2,21,22^.

Wheat is most significant among cereal crops, extensively farmed by smallholder farmers in a wide range of altitudes under rainfed areas and provide food for almost half of the global population. It is ranked second in total production but first in the area under cultivation^23,24^. The demand for wheat is expected to increase by 60% by 2050 to feed the growing world population, which will reach nine billion. The worldwide average wheat yields must grow at a pace of at least 1.6% per year, up from the current 1% to meet this requirement^25^. The principal causes of loss of wheat production are abiotic factors such as drought, salinity, and heat stress rather than biotic factors^26^. Drought stress is a physical phenomenon in environment which has a significant impact on the yield of wheat. Globally, drought spells are becoming more frequent and severe due to rising temperatures and fluctuating precipitation while changing climate is exacerbating the situation even more^27,28^. In addition, its frequency and severity are likely to intensify due to global warming in the coming days^1,29^. A variety of researchers have demonstrated that wheat is vulnerable to osmotic stress^1^, resulting in yield losses of up to 90% depending on the genotype, plant growth stage, and the intensity and duration of drought spells^16,27,30–32^.

In the current study, the quantitative expression patterns of *TaARGOS* homoeologous genes in inevitable drought tolerant and sensitive bread wheat genotypes were quantified to determine the resilience pathway against PEG-induced dehydration and response of studied genes at various time points.

## Materials and methods

### Identification of target homoeologous genes and primers designing

This study used three homoeologous *TaARGOS* genes (*TaARGOS-A*, *TaARGOS-B*, and *TaARGOS-D*) for quantitative expression profiling as target genes. At the same time, *DREB3* was used as a marker gene to distinguish the pervasiveness of osmotic stress in treated wheat seedlings^33^. “Primer Blast” tool from NCBI database (https://www.ncbi.nlm.nih.gov) was employed to generate the sequence of primers for *TaARGOS* and *DREB3* genes and were reinforced by employing online software “Primer 3 Version-0.4.0” (available at https://www.bioinfo.ut.ee). A reported portion of the wheat *β-actin* gene was also amplified as an endogenous or internal control to normalize the target genes^2,16^. The designed primers were prepared commercially (Eurofins MWG Operon, United States of America). The genomic sequences of the three homologs of *TaARGOS*, *DREB3* and endogenous *β-actin* genes were aligned, and their primers were designed to target comparable genomic loci (Table 1).

**Table 1:** Primer amplification characteristics of the target, marker, and endogenous genes for qRT-PCR in bread wheat.

Primer	Gene Bank accession number	Sequence information	Tm	GC%	Amplicon size (bp)	CDS sequence size (bp)
*TaARGOS-A*	KX768731	F: 5′-GATCATCTTCCACCACCATCTC-3′	60.72	50	117	2089
		R: 5′-GCACCTACATGGGTGTTCTT-3′	57.51	50		
*TaARGOS-B*	KX768732	F: 5′-GATTTGGAGGAGAGGGTGTTC-3′	59.93	55	120	2047
		R: 5′-GCGTGGACAATCAAGCATAAG-3′	60.65	50		
*TaARGOS-D*	KX768733	F: 5′-GCTGATCTGCACTCACCAAA-3′	59.99	50	156	2057
		R: 5′-CAACCTTCTTGTCAGCAGCA-3′	60.18	50		
*DREB3*	AY781349	F: 5′-GGCATGCTGCAGTCTGATTA-3′	59.98	50	156	1325
		R: 5′-AAGCCGACCAAACACCATAG-3′	59.99	50		
*β-actin*	AK457930.1	F: 5′-GGAATCCATGAGACCACCTAC-3′	58.31	52	128	1527
		R: 5′-GACCCAGACAACTCGCAAC-3′	59.25	58		

### Plant materials

Three drought-tolerant (Pakistan-2013, NARC-2009, and NR-499) and three drought-sensitive (Borlaug-2016, NR-514 and NR-516) wheat genotypes were employed in this investigation, acquired from Wheat Program, Crop Science Institute (CSI), National Agricultural Research Centre (NARC), Islamabad. These contrasting bread wheat genotypes were identified in our earlier findings based on physiological, biochemical and morphological traits as reported^27^. The detail of used plant material is presented in Table 2. The study was performed in the laboratory and glasshouse of the National Institute for Genomics and Advanced Biotechnology (NIGAB), NARC, Islamabad, Pakistan. All the experiments were performed in accordance with relevant guidelines and regulations.

**Table 2:** List and description of bread wheat genetic material.

S. no.	Genotype	Source	Characteristics
**Drought tolerant genotypes**			
1	Pakistan-2013	WP–CSI–NARC	Low rainfed area, stem, leaf and yellow rust resistance
2	NARC-2009	WP–CSI–NARC	Drought tolerant, yellow and leaf rust resistance
3	NR-499	CIMMYT/WP–NARC	Advance line (CIMMYT)
**Drought sensitive genotypes**			
4	Borlaug-2016	WP–CSI–NARC	Rainfed areas, UG-99, leaf and yellow rust resistance
5	NR-514	CIMMYT/WP–NARC	Advance line (CIMMYT)
6	NR-516	CIMMYT/WP–NARC	Advance line (CIMMYT)

*WP* Wheat Program, *CSI* Crop Science Institute, *NARC* National Agricultural Research Centre, Islamabad, *CIMMYT* International Maize and Wheat Improvement Center.

### PEG treatment for dehydration and osmotic stress

Wheat seeds were surface sterilized with 4% sodium hypochlorite and rinsed thricely with distilled water. The seed were then allowed to germinate in growth chamber (SANYO MLR 350-H) for 8 (± 2) days at a standard day and night temperature of 22 ± 2 ℃ and 50% relative humidity^34^. At mid-canopy, light intensity was regulated at 400 µmoles m^−2^ s^−1^. The photoperiod was also controlled by maintaining 16 h for light and 8 h for dark. Young wheat seedlings at two to three leaf stage of all six parental lines were transferred into two different solutions; one of which included PEG-6000 @ 25% for the induction of osmotic dehydration. In contrast, the control group of seedlings was transferred to distilled water^16^. The quantitative expression profiling was conducted at five different time points: 0 (exogenous control), 2, 4, 6, and 12 h.

### Sample assortment and total RNA extraction

For the expression analyses, seedlings from the control and stress groups were collected. After being exposed to PEG-induced dehydration, the adolescent leaves were frozen with liquid nitrogen immediately to preserve all their proteomics at a particular stage and then kept at a temperature of − 80 °C until needed. Total RNA was extracted from the leaves of control and stressed seedlings using TRIzol™ Reagent (Invitrogen, Shanghai) by grinding frozen leaf samples in liquid nitrogen followed by the manufacturer’s instructions^35^.

### Measurement and equilibration of total RNA

Total RNA quality and concentrations were determined using a spectrophotometer (NanoDrop ND-1000, Wilmington, USA) with the Optimal Density (OD) group at 260/280 nm and Nucleic Acid Concentration in ng/l. RNA equilibration was followed using nuclease-free water. The electrophoresis on a 2% agarose gel was used to validate the presence and purity of RNA.

### Synthesis of 1st strand of cDNA

The First Strand cDNA Synthesis RevertAid (ThermoFisher Scientific) Kit was utilized for cDNA synthesis as per the manufacturer’s directions, as 0.1–5 ng RNA template, 1 μl of Oligo dT_18_ anchor primer, and 12 μl nuclease-free water were merged and incubated for 5 min at 65 °C. The samples were moved to the ice after incubation, and reaction buffer, RNase inhibitor, a mixture of dNTP, and reverse transcriptase (M-MuLV RT) were added to get the volume up to 20 μl. The samples were then incubated at 42 °C for 80 min, 70 °C for 5 min and the RT-PCR reaction was stopped at 4 °C. The synthesized cDNA of all wheat samples was kept at − 20 °C until further use.

### Real-time qPCR procedure

The Maxima SYBR Green qPCR Master Mix (ThermoFisher Scientific) was employed for real-time expression profiling according to the manufacturer’s recommendations. The 12.5 μl Maxima SYBR Green qPCR Master Mix (2×), 0.3 μl forward primer, 0.3 μl reverse primer, ≤ 500 ng cDNA template and nuclease-free water were mixed to raise total volume upto 25 μl. A preliminary phase at 95 °C for 30 s, followed by 40 cycles at 95 °C for 15 s and 60 °C for 35 s, with a definitive extension at 72 °C of 7 min. Reaction tubes were inserted in the cycler, and then the program for quantitative expression analysis on Mic qPCR (Bio-Molecular System) was run. Ultimately, the resultant Ct data were obtained for expression analysis.

### Quantitative (qPCR) expression data analysis

Each expression profile was analyzed and authenticated with three biological and three technical replicates for all qRT-PCR analyses. The Quantification Cycle (Cq) or Threshold Cycle (Ct) data for exogenous control from non-treated plant samples, while endogenous control from *β-actin* was obtained. The double delta Ct value was used to calculate relative gene expression or expression fold for each sample^36^. Relative fold expression was recorded using Ct value experimental (target) and Ct value control genes.

$$ {\text{Double delta Ct }} = { 2}^{{( - \, \Delta \Delta {\text{Ct}})}} . $$

### Statistical analysis

The data was analyzed for Factorial Completely Randomized Design (CRD) Analysis of Variance (ANOVA) under General Linear Model (GLM)^37^, followed by Tukey’s Honestly Significant difference (HSD) at 5% probability level in order to determine mean comparisons, using “IBM Statistical Program for Social Science (SPSS Version 22)” software.

### Cluster analysis based heatmapping for genes × genotypes

Heatmapping for genes × genotypes interaction was used in conjunction with cluster analysis at certain timepoints to depict all of the genes with all of the genotypes and timepoints investigated simultaneously based on Euclidean distances using R-Studio software (R Development Core Team 2020) by employing *hclust* and *pheatmap-package*^38^.

### Ethical approval

This article does not contain any studies with human participants or animals performed by any of the authors.

## Results and discussion

Gene expression profiling quantifies mRNA levels at certain circumstances, revealing the pattern of genes expressed by a cell at the transcription level.

### Expression analysis of *DREB3* gene under osmotic stress

**Figure 1:**
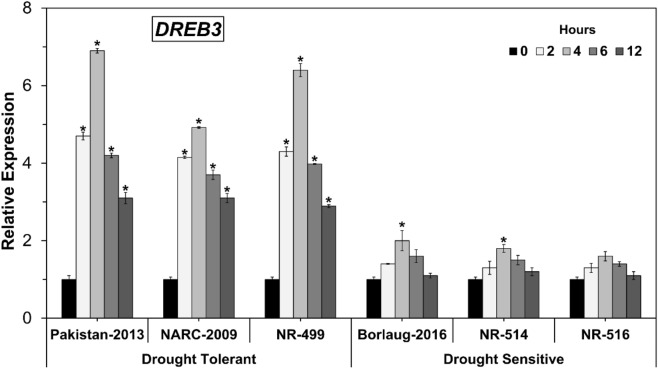
*DREB3* expression levels in drought-tolerant and sensitive wheat genotypes. (Asterisk indicates P < 0.05).

As a fundamental family of the transcription factor, *DREB* assumes a significant part in countering abiotic stresses (Niu et al.^33^). The expression profiling of the wheat *DREB3* gene was first examined after PEG treatment for various timespans such as 0, 2, 4, 6, and 12 h. In drought-tolerant wheat genotypes (NR-499, NARC-2009, and Pakistan-2013), the expression of the *DREB3* gene was incline significantly at 2 h (> fourfold), peaked at 4 h (6–7 folds) and then started to decline after 6 h of moisture stress (Fig. 1). In any case, it generally diminished after 6 h and recorded the most negligible and least expression at 12 h during this study. These results suggest that *DREB3* is an early drought-responsive gene.

Among drought-tolerant wheat genotypes, Pakistan-2013 showed the most elevated expression pattern of the *DREB3* gene followed by NR-499 and NARC-2009. Although, drought-sensitive genotypes showed similar increase–decrease trend, but the significant change (increase) in expression of *DREB3* was only observed in Borlaug-2016 (twofold) and NR-514 (1.8-fold) after 4 h of osmotic stress while NR-516 did not show any significant change in *DREB3* expression (Fig. 1). In this way, moderately higher expression of the *DREB3* gene in drought-tolerant genotypes suggests that the *DREB3* is involved in PEG-induced dehydration resilience in bread wheat. In addition, it also shows that drought-tolerant wheat genotypes have the hereditarily genetic potential to endure dry spells. Several previous studies also described that different abiotic stresses trigger the expression pattern of *TaDREB3* and dehydrin genes as a counter mechanism, as observed in this study in case of *TaDREB3*^33,39^.

### Expression analysis of *TaARGOS* genes under osmotic stress

*ARGOS* genes are engaged with different formative and stress reactive mechanisms in plants^16,18^. Earlier findings showed that the *ARGOS* genes of Arabidopsis and maize alleviate disease resistance and can be set off by different chemicals and hormones. In wheat, the expression of *TaARGOS* genes have been determined for in organ improvement and stress response^16^. Subcellular restriction of *ARGOS-D* protein indicated that it is limited to the endoplasmic reticulum^16^. The expression level of wheat *TaARGOS* genes was concentrated after osmotic pressure at various time stretches to determine whether *ARGOS* genes influence to improve resilience against PEG-induced dehydration in distinguished wheat lines. Distinctive expression levels were portrayed for *TaARGOS-A*, *TaARGOS-B*, and *TaARGOS-D*, articulating that these homeologs can perform important functions at various phases of growth and development.

### Expression analysis of *TaARGOS-A* gene under osmotic stress

**Figure 2:**
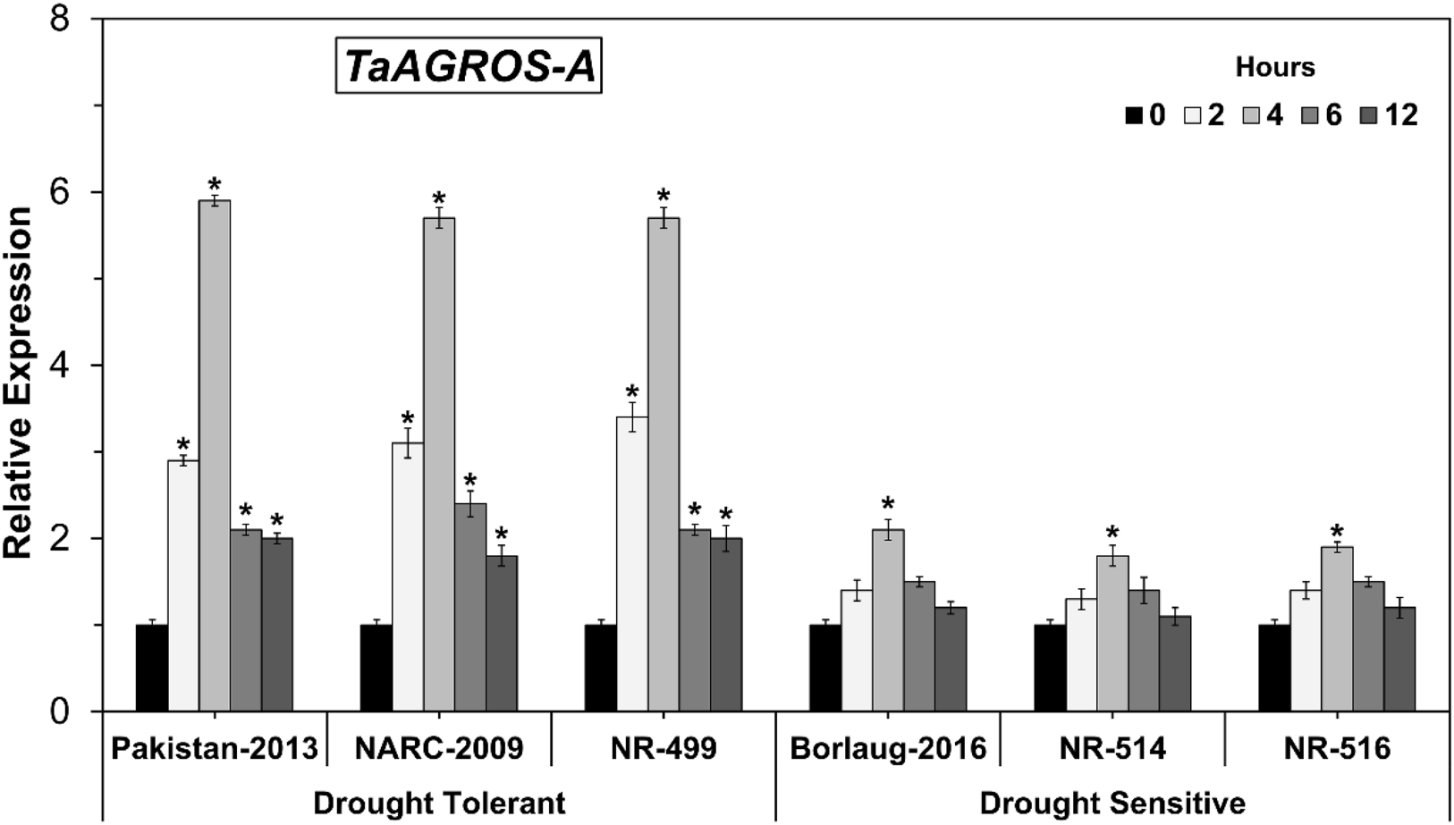
*TaARGOS-A* expression levels in drought-tolerant and sensitive wheat genotypes. (Asterisk indicates P < 0.05).

*TaARGOS-A* is the first homoeologous gene of the *TaARGOS* gene family, located on chromosome 4A in bread wheat^16^. In present study, initially up to 4 h of osmotic stress the expression of *TaARGOS-A* increased significantly, followed by a continuous decline till 12 h of osmotic stress in all the studied genotypes (Fig. 2). Importantly, all three drought tolerant genotypes showed markedly higher expression of *TaARGOS-A* than drought sensitive genotypes. For instance, the highest mRNA transcription of the *TaARGOS-A* gene was observed in Pakistan-2013, that showed 5.9 fold increase in gene expression at 4 h, followed by NARC-2009 and NR-499 at the same time point. In contrast, the drought sensitive genotypes did not show any significant change (except slight increase or decrease across the timepoint) in *TaARGOS-A* expression, except the significant increase after 4 h of osmotic stress which was highest in Borlaug-2016, followed by NR-516 and NR-514. These outcomes depicted that *TaARGOS-A* is a drought-responsive gene and engaged in dehydration resilience in wheat by aggregating in greater extents in the drought-tolerant genotypes under water limiting conditions. Similar findings were documented in Arabidopsis, bread wheat and maize, respectively^2,16,19^.

### Expression analysis of *TaARGOS-B* gene under osmotic stress

**Figure 3:**
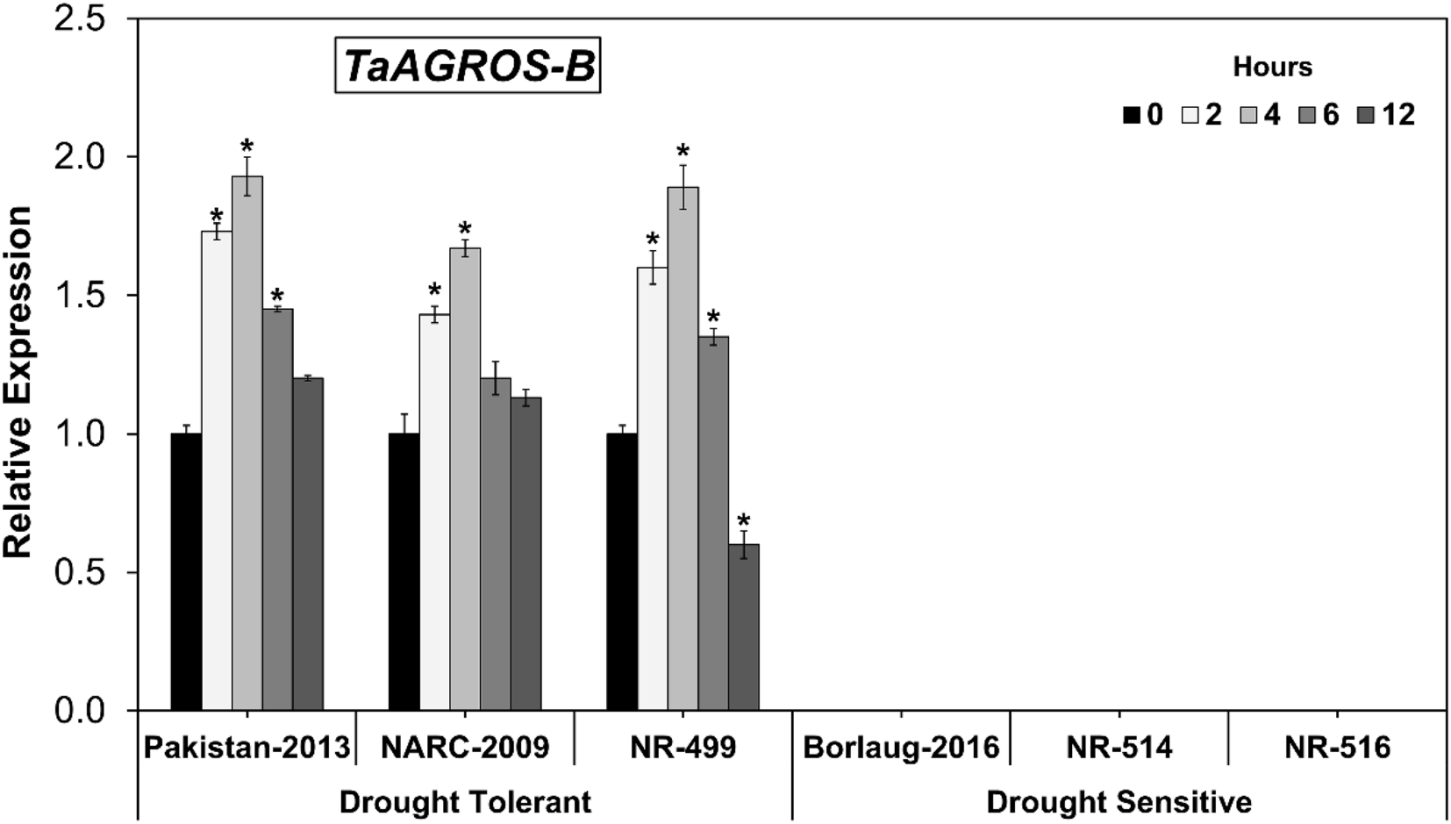
*TaARGOS-B* expression levels in drought-tolerant and sensitive wheat genotypes. (Asterisk indicates P < 0.05).

The expression pattern of *TaARGOS-B* homeolog was likewise concentrated in drought-tolerant and sensitive wheat genotypes. Outcomes publicized that the expression level of *TaARGOS-B* was relatively lower in wheat genotypes under moisture deficient conditions contrasted with *TaARGOS-A* and *TaARGOS-D*. The expression of the *TaARGOS-B* gene was identified distinctly in drought-tolerant wheat genotypes; however, among drought-sensitive genotypes, no indication of gene expression was identified (Fig. 3). The explanation could be because of the mutation in the promoter region of this gene in such drought-sensitive genotypes, as detailed beforehand in wheat^16^. Since it was expressed distinctly only in three drought-tolerant wheat genotypes, therefore, this gene could have a significant feature in terms of its improved drought tolerance potential in wheat. Further research to discover the SNP variation for the advancement of *TaARGOS-B* allied markers would enormously help contrive quick and fast molecular markers for expedient crop breeding^40,41^.

Results illustrated that the expression pattern of the *TaARGOS-B* gene was actuated at 2 h following moisture stress and crested at the most elevated at 4 h of osmotic stress. Subsequently, mRNA accretion dynamically diminished, as it commenced to decrease at 6 h and reached at least expression fold at 12 h. The wheat genotypes Pakistan-2013 and NR-499 displayed the most remarkable expression of the *TaARGOS-B* by around two-folds compared to NARC-2009 (Fig. 3). Moreover, *TaARGOS-B* unveiled no or zero-fold expression at all studied timepoints among all three drought sensitive wheat genotypes (Fig. 3).

### Expression analysis of *TaARGOS-D* gene under osmotic stress

**Figure 4:**
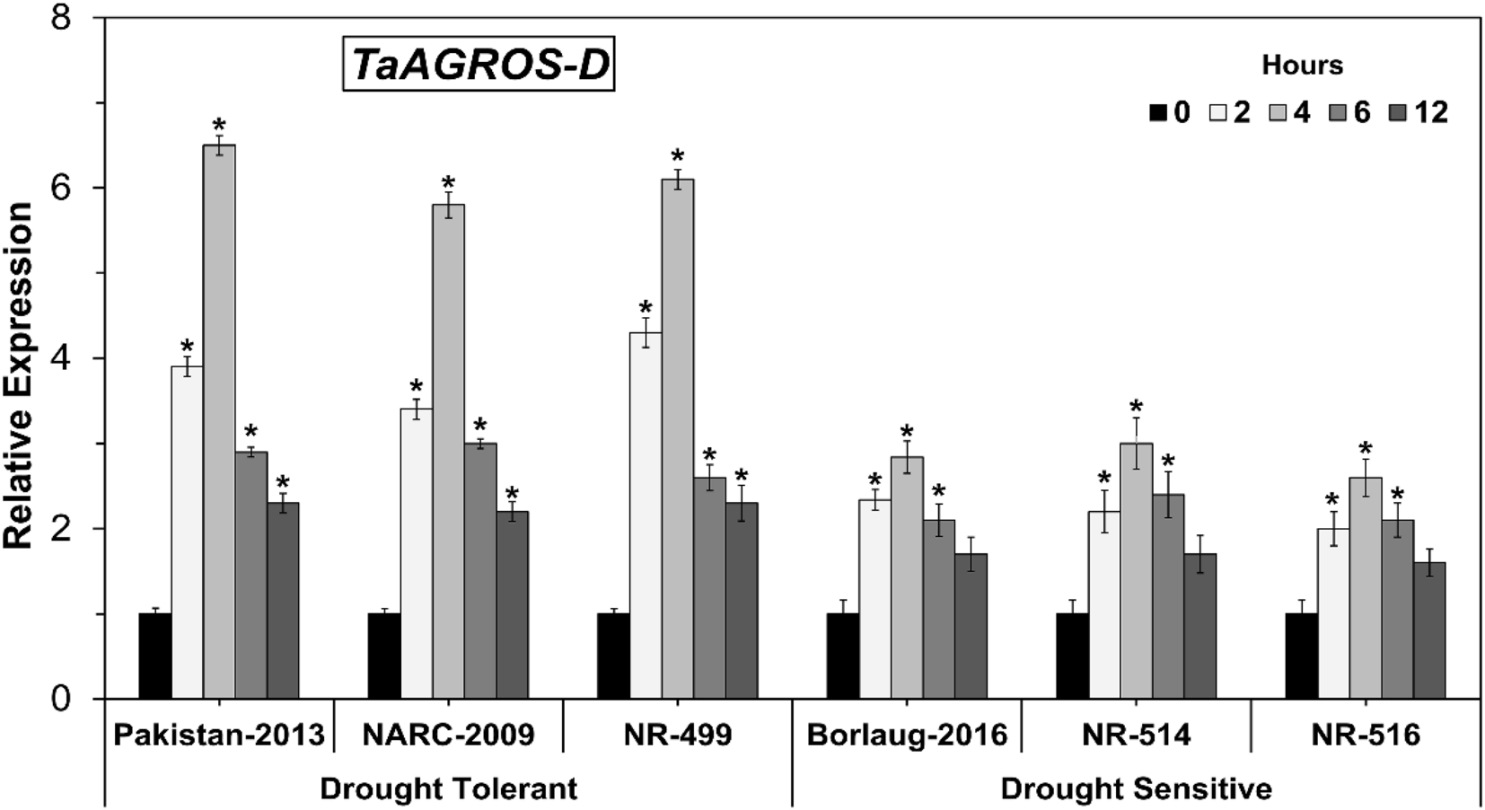
*TaARGOS-D* expression levels in drought-tolerant and sensitive wheat genotypes. (Asterisk indicates P < 0.05).

Finally, the expression pattern of the *TaARGOS-D* gene in drought-tolerant and sensitive genotypes was investigated in increasing osmotic stress. The *TaARGOS-D* gene expression was determined in drought-tolerant genotypes to be considerably higher than in the sensitive group of genotypes, indicating the genetic potential of a tolerant group of genotypes (Fig. 4). The expression was higher at 2 and 4 h, but it began to decline afterward, indicating that sufficient protein was accumulated to cope with certain stress levels^16^. In addition to the drought-tolerant genotypes, Pakistan-2013 showed the greatest transcription by 6.5 folds of the *TaARGOS-D* gene at 4 h, followed by NR 499 and NARC-2009.

Conversely, the expression of the *TaARGOS-D* gene was stimulated 3–4 folds in drought sensitive genotypes, substantially implying that the transcription of *TaARGOS-D* articulated drought response in wheat (Fig. 4) substantially. In terms of the expression pattern of *TaARGOS-D* homolog, the drought sensitive genotypes showed non-significant variabilities. The *TaARGOS-D* gene is responsive to different abiotic stresses, notably drought and salt tolerance, and plays a significant role in many plants growth and developmental processes by activating auxin-based regulatory pathways, as previously described^16^. As a result, it might be useful in developing drought-tolerant wheat cultivars for arid to semi-arid climatic conditions.

### Genes × genotypes cluster-based heatmapping

A heatmap was graphed to showcase the comparative levels and overall performance of all target genes and wheat genotypes examined at different periods. Predicted gene expression profiles as a heatmap has recently appeared as an excellent and popular technique to expediently visualize the expression pattern^11,15,42^.

**Figure 5:**
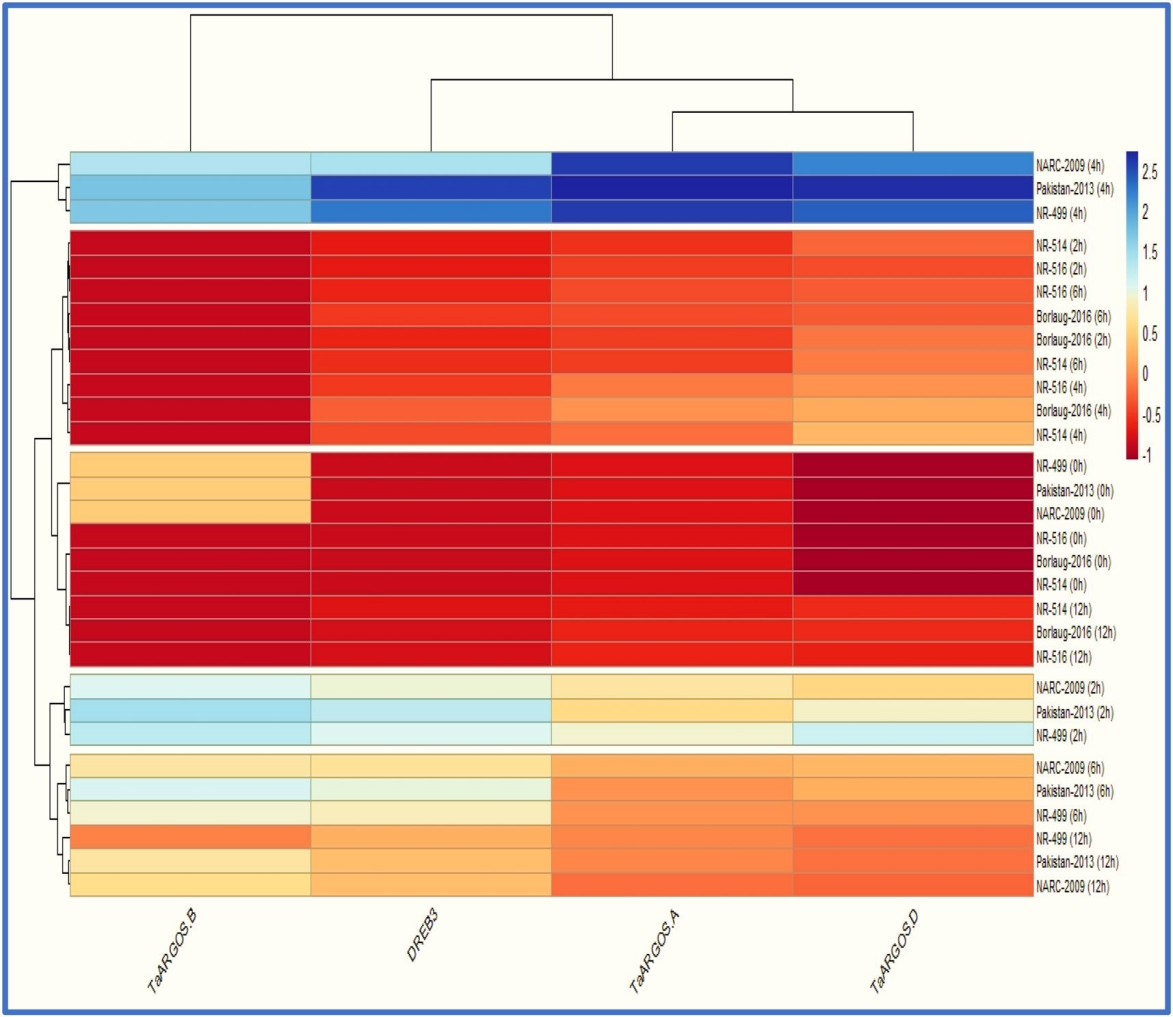
Expression heatmap and cluster analysis for *DREB3* and *TaARGOS* genes among wheat genotypes.

Furthermore, clustering in the heatmap further enables the grouping of genes and samples with similar expression patterns much easier^15^. As a result, cluster analysis-based heatmapping for gene × genotypes association was used to simultaneously display the trend and tendencies of both genes and genotypes^1^. Corresponding to the color scale illustrated by the color strip, the positive darker scale represents drought-tolerant genotypes at a given osmotic stress timepoint. In contrast, the negative darker color strip represents low-expressing vulnerable genotypes (Fig. 5). Likewise, when the color strength diminishes, the specific strip-line exhibits a subtle and modest expression pattern on both positive and negative sides.

The expression-heatmap, along with cluster analysis, identified two main clades and five sub-clades among the six wheat genotypes examined at certain time intervals (Fig. 5). The first clade was visualized individually, with three drought-tolerant wheat genotypes, i.e., Pakistan-2013, NARC-2009, and NR-499, only at 4 h of timepoint with darker positive color-strip. It suggested that all of the studied genes publicized significantly greater expression for such genotypes at a particular timepoint by contrast with the rest of the genotypes and timepoints.

The second clade includes control samples from drought-tolerant and drought-sensitive genotypes. All four studied genes portrayed a decreased expression pattern in this group. At 2, 6, and 12 h, the other sub-groups of the second clade revealed the existence of drought-tolerant genotypes. All genes in this group have expression profiles that vary from low to medium. The wheat variety Pakistan-2013 most efficiently outperformed in terms of mRNA transcription for *TaARGOS-D*, *TaARGOS-A*, and *DREB3* at 4 h, followed by NARC-2009 and NR-499 for *TaARGOS-A* at a similar timepoint.

Furthermore, the cluster analysis revealed that the *TaARGOS-A* and *TaARGOS-D* genes cluster together and provide almost identical results. Because of the distinct expression pattern, the *DREB3* gene was placed in a different category than the other three *TaARGOS* homoeologous genes. The *TaARGOS-B* gene expression pattern was also found to be divergent from the *TaARGOS-A* and *TaARGOS-D* genes for a tolerant group of wheat genotypes. It was found to be the least expressive gene in the tolerant group of genotypes. Contrastingly, no expression was detected in drought sensitive genotypes. Due to this, the *TaARGOS-B* gene in the cluster-based heatmap was separated from the other two homologs of *TaARGOS*.

These findings, when considered collectively, give important information on valuable selection indicators for wheat dehydration resistance. Drought tolerance in wheat is associated with several critical agronomic and physiological characteristics for efficient selection, as reported by many former researchers^2,10^. Additionally, according to the findings, drought tolerance in wheat genotypes under study is also influenced by the *DREB3* and *TaARGOS* genes at the molecular level. Finally, based on the seedling stage, it can be concluded that Pakistan-2013, NR-499, and NARC-2009 have proved to be the most drought-tolerant wheat genotypes. These identified bread wheat genotypes may further be employed as drought-tolerant parental lines. Additionally, molecular characterization of *ARGOS* homoeologous genes in other elite and commercial cultivars of bread wheat and other crops, that can be most proficient approach for various breeding programs to mitigate intensifying climatic variability for arid and semi-arid regions of the world.
